# Predicting tumor mutation burden and VHL mutation from renal cancer pathology slides with self‐supervised deep learning

**DOI:** 10.1002/cam4.70112

**Published:** 2024-08-21

**Authors:** Qingyuan Zheng, Xinyu Wang, Rui Yang, Junjie Fan, Jingping Yuan, Xiuheng Liu, Lei Wang, Zhuoni Xiao, Zhiyuan Chen

**Affiliations:** ^1^ Department of Urology Renmin Hospital of Wuhan University Wuhan Hubei China; ^2^ Institute of Urologic Disease Renmin Hospital of Wuhan University Wuhan Hubei China; ^3^ Centre for Reproductive Science Renmin Hospital of Wuhan University Wuhan Hubei China; ^4^ University of Chinese Academy of Sciences Beijing China; ^5^ Trusted Computing and Information Assurance Laboratory Institute of Software, Chinese Academy of Sciences Beijing China; ^6^ Department of Pathology Renmin Hospital of Wuhan University Wuhan Hubei China

**Keywords:** artificial intelligence, attention‐based multiple instance learning, clear cell renal cell carcinoma, self‐supervised learning, tumor mutation burden, VHL mutation

## Abstract

**Background:**

Tumor mutation burden (TMB) and VHL mutation play a crucial role in the management of patients with clear cell renal cell carcinoma (ccRCC), such as guiding adjuvant chemotherapy and improving clinical outcomes. However, the time‐consuming and expensive high‐throughput sequencing methods severely limit their clinical applicability. Predicting intratumoral heterogeneity poses significant challenges in biology and clinical settings. Our aimed to develop a self‐supervised attention‐based multiple instance learning (SSL‐ABMIL) model to predict TMB and VHL mutation status from hematoxylin and eosin‐stained histopathological images.

**Methods:**

We obtained whole slide images (WSIs) and somatic mutation data of 350 ccRCC patients from The Cancer Genome Atlas for developing SSL‐ABMIL model. In parallel, 163 ccRCC patients from Clinical Proteomic Tumor Analysis Consortium cohort was used as independent external validation set. We systematically compared three different models (Wang‐ABMIL, Ciga‐ABMIL, and ImageNet‐MIL) for their ability to predict TMB and VHL alterations.

**Results:**

We first identified two groups of populations with high‐ and low‐TMB (cut‐off point = 0.9). In two independent cohorts, the Wang‐ABMIL model achieved the highest performance with decent generalization performance (AUROC = 0.83 ± 0.02 and 0.8 ± 0.04 in predicting TMB and VHL, respectively). Attention heatmaps revealed that the Wang‐ABMIL model paid the highest attention to tumor regions in high‐TMB patients, while in VHL mutation prediction, non‐tumor regions were also assigned high attention, particularly the stromal regions infiltrated by lymphocytes.

**Conclusions:**

Our results indicated that SSL‐ABMIL can effectively extract histological features for predicting TMB and VHL mutation, demonstrating promising results in linking tumor morphology and molecular biology.

## Graphical abstract

Tumor mutation burden (TMB) and VHL mutation play a crucial role in the management of patients with clear cell renal cell carcinoma (ccRCC), but the time‐consuming and expensive high‐throughput sequencing methods severely limit their clinical applicability. Our aim was to develop a self‐supervised attention‐based multiple instance learning (SSL‐ABMIL) model to predict TMB and VHL mutation status from hematoxylin and eosin‐stained histopathological images. Our results indicated that SSL‐ABMIL can effectively extract histological features for predicting TMB and VHL mutation, demonstrating promising results in linking tumor morphology and molecular biology.

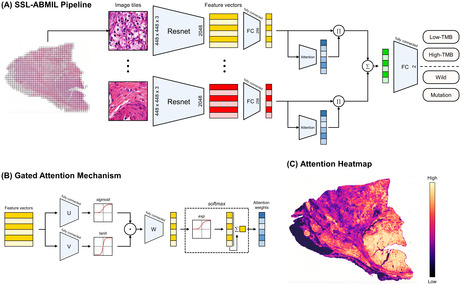

## 1 INTRODUCTION

Clear cell renal cell carcinoma (ccRCC) is the most commonly diagnosed histological subtype of renal cell carcinoma and accounts for the majority of kidney cancer‐related deaths (75%).^1^ Immunotherapy targeting the tyrosine kinase inhibitors and immune checkpoint inhibitors, exemplified by CLAT‐4 and PD‐1/PD‐L1, have shown great promise in the treatment of ccRCC.^2^, ^3^ However, durable benefits are limited to a minority of patients, highlighting the clinical need to identify better predictive biomarkers. Tumor mutation burden (TMB) is an emerging immunotherapy sensitivity biomarker that has been demonstrated to be significantly associated with immunotherapy response.^4^, ^5^ In fact, metastasis and treatment resistance are generally driven by intratumoral heterogeneity, requiring comprehensive tumor sequencing to reliably capture all mutational events, which is clinically impractical.^6^, ^7^ Therefore, predicting TMB and key mutations in ccRCC is a critically needed research task due to its high clinical relevance: first, TMB is a strong predictor of response to cancer immunotherapy‐related prognosis;^4^, ^5^, ^8^ second, TMB and key mutations such as VHL are highly associated with clinical outcomes.^9^, ^10^

Digital histopathology slides stained with hematoxylin and eosin (H&E) contain rich diagnostic and prognostic information, which can be effectively quantified and explored using artificial intelligence techniques, particularly deep learning (DL).^11^ DL has proven to be effective in tumor pathology diagnosis,^12^ prognosis prediction,^13^ molecular subtyping^14^ and predicting clinically relevant biomarkers,^15^ greatly advancing the field of computational pathology. In ccRCC, DL‐based prediction of biomarkers from H&E‐stained tissue slides has been reported for certain mutations, such as BAP1,^16^ and for some smaller studies on TMB.^17^, ^18^ However, various issues related to clinical applicability remain unresolved. The most significant concern with existing DL algorithms is the problem of generalizability.^19^ Typically, noticeable performance degradation is observed when models trained internally are deployed on external cohorts. Improving model prediction performance while maintaining generalizability on external datasets is crucial for clinical utility. The second issue is model interpretability. Understanding the predictive patterns learned by DL models contributes to the development of new morphological knowledge, that is, which morphological features are closely associated with the predictions. The third issue is the applicability of the model, that is applying the DL models to other key mutations beyond BAP1. However, VHL, as the most frequent mutation gene, has been rarely investigated for its alterations in multiple patient cohorts.

In computational pathology, the most widely used predictive method is training multiple instance learning (MIL) models by tiling image blocks from digitized whole slide images (WSIs).^20,21^ Each image tile inherits the label of the WSI, necessitating the aggregation of tile‐level predictions into WSI‐level predictions. A common approach is to employ transfer learning model pretrained on ImageNet (ImageNet‐MIL) and aggregate predictions using average or max pooling functions.^22^, ^23^ Subsequently, Ilse et al.^24^ proposed attention‐based multiple instance learning (ABMIL) using gated attention mechanism. The image feature extractor in ABMIL can be pretrained using self‐supervised learning (SSL), demonstrating superior performance compared to the ImageNet‐MIL approach.^25^ Increasing evidence suggests that SSL and ABMIL are crucial components in computational pathology, although their integration has not been extensively validated for predicting tumor heterogeneity in ccRCC. The lack of independent validation remains a significant obstacle to clinical application.^19^

Hence, in this study, we adopted the ImageNet‐MIL approach as the baseline model and trained ABMIL models with two SSL‐encoder (Wang^26^ and Ciga^27^) for predicting TMB and VHL mutation status in ccRCC. Our findings from two independent cohorts demonstrated that DL‐based neural network models can identify the intratumoral heterogeneity of ccRCC, including TMB and VHL mutation, from routine H&E‐stained WSI. The ABMIL approach with SSL‐trained feature extractors outperforms the ImageNet‐MIL method and exhibits better generalization to external cohort.

## 2 MATERIALS AND METHODS

### 2.1 Patient cohorts

In this study, we employed two independent cohorts of ccRCC patients, along with corresponding H&E‐stained WSIs and mutation status. Both cohorts consisted exclusively of formalin‐fixed, paraffin‐embedded samples, which are standard in clinical practice and better preserve tissue morphology compared to frozen sections.

The Cancer Genome Atlas (TCGA) Cohort: This cohort served primarily as the training data for our models. It comprised 519 complete H&E‐stained WSIs of ccRCC, with corresponding genomic sequencing data sourced from the Pan Cancer Atlas study.^28^ These WSIs and clinico‐pathological information can be directly downloaded from the TCGA‐KIRC project on the NIH GDC Data Portal, and the corresponding genomic profiles are accessible from the TCGA‐KIRC Pan Cancer Atlas project on the cBioPortal portal (RRID: SCR_014555). Among the total of 519 samples, we excluded samples with damaged H&E‐stained images and missing mutation data, resulting in 356 available WSIs for this study.

Clinical Proteomic Tumor Analysis Consortium (CPTAC) Cohort: This cohort was primarily used to validate our models. It comprised 524 complete H&E‐stained WSIs of ccRCC. These WSIs, along with clinico‐pathological information and corresponding genomic profiles, were downloaded from the CPTAC Data Portal. Among the total of 222 samples, we excluded samples with damaged H&E images and missing mutation data, resulting in 232 available WSIs for this study.

We constructed our model using the TCGA cohort, which comprised 356 WSIs from a total of 350 ccRCC patients. Additionally, 232 WSIs from 163 ccRCC patients in the CPTAC cohort were utilized as independent external validation set. Table 1 presents the baseline characteristics of the TCGA and CPTAC cohorts. The detailed data distribution is provided in Additional file 1: Table S1.

**TABLE 1:** Clinico‐pathological features of both TCGA and CPTAC cohorts.

	TCGA	CPTAC
Number of patients	350	163
WSI format	SVS	SVS
TMB		
Low (≤ 0.9)	109 (31.14%)	30 (18.4%)
High (>0.9)	241 (68.86%)	133 (81.6%)
VHL		
Mutation	142 (40.57%)	113 (69.33%)
Wild type	208 (59.43%)	50 (30.67%)
Age (years)	59.98 (±12.06)	60.87 (±12.12)
Gender		
Female	127 (36.29%)	55 (33.74%)
Male	223 (63.71%)	108 (66.26%)
pT stage		
pT1	194 (55.43%)	50 (30.67%)
pT2	49 (14.00%)	11 (6.75%)
pT3	102 (29.14%)	36 (22.09%)
pT4	5 (1.43%)	3 (1.84%)
pTx	0 (0%)	63 (38.65%)
pN stage		
pN0	149 (42.57%)	26 (15.95%)
pN1	13 (3.71%)	3 (1.84%)
pNx	188 (53.72%)	134 (82.21%)
pM stage		
pM0	281 (80.29%)	26 (15.95%)
pM1	40 (11.43%)	3 (1.84%)
pMx	29 (8.28%)	134 (82.21%)
pTNM stage		
Stage I	188 (53.71%)	73 (44.79%)
Stage II	40 (11.43%)	18 (11.04%)
Stage III	75 (21.43%)	38 (23.31%)
Stage IV	46 (13.14%)	18 (11.04%)
Missing	1 (0.29%)	16 (9.82%)
Histologic grade		
G1	12 (3.43%)	10 (6.13%)
G2	149 (42.57%)	79 (48.47%)
G3	135 (38.57%)	56 (34.36%)
G4	47 (13.43%)	18 (11.04%)
Gx	7 (2.00%)	0 (0%)
Survival status		
Alive	258 (73.71%)	122 (74.85%)
Dead	92 (26.29%)	25 (15.34%)
Not Reported	0 (0%)	16 (9.81%)
Overall survival (years)	3.54 (± 2.70)	2.09 (± 1.69)

### 2.2 Identification of TMB and VHL mutation

First, we collected somatic mutation data from the TCGA and CPTAC cohorts and performed calculation of TMB scores. TMB scores were calculated using the Maftools packages.^29^ The maximally selected rank statistics method^30^ was employed to identify the optimal stratification threshold, dividing patients in the TCGA cohort into high‐ and low‐TMB groups, which was subsequently extended to the CPTAC cohort. We selected the VHL gene,^31^ which exhibits the highest mutation frequency and significant clinical relevance, for analyzing tumor heterogeneity in ccRCC.

### 2.3 WSI preprocessing

All H&E‐stained WSIs were downsampled to 20× magnification for analysis. The tissue regions of the WSIs were segmented using the Otsu algorithm,^32^ followed by tiling the segmented foreground portions to obtain image tiles of 448 × 448 pixels with a length of 224 μm. RGB thresholding was applied to filter out potentially mixed background tiles, ensuring that the remaining images contained at least 80% tissue content. Color normalization of all image tiles was performed using the method proposed by Vahadane.^33^

### 2.4 TMB and VHL mutation prediction from WSI

In this study, we compared the results obtained using two different DL methods: ImageNet‐MIL and SSL‐ABMIL. Both methods aim to solve a binary classification problem by predicting WSI‐level labels from a collection of individual image tiles.

The ImageNet‐MIL method^23^, ^24^ employs a pretrained model based on ImageNet as a feature extractor. It aggregates tile‐level predictions using average or maximum pooling functions to obtain WSI‐level predictions. This approach is currently popular and has shown good performance in mutation prediction, but it is not perfect for external cohorts.^16^

**FIGURE 1:**
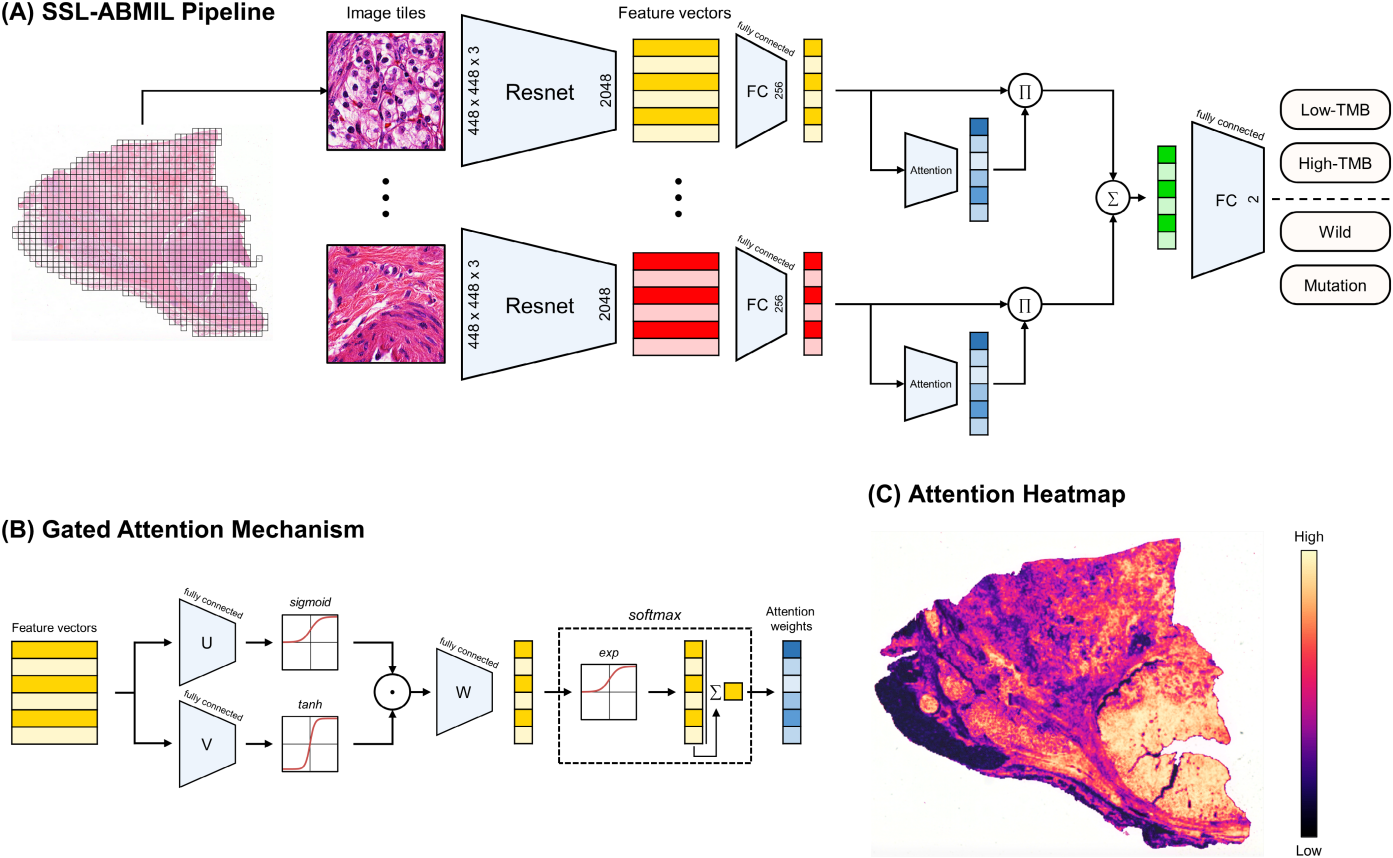
Schematic workflow of the study. (A) The strategy of the SSL‐ABMIL model for predicting TMB and VHL gene mutation status from H&E‐stained images. (B) Graphical explanation of the gated attention mechanism. (C) Attention weights generated by the SSL‐ABMIL model to generate heatmaps, revealing the connection between morphological features and molecular biology. SSL, self‐supervised learning; ABMIL, attention‐based multiple instance learning; TMB, tumor mutation burden; H&E, hematoxylin and eosin.

In the SSL‐ABMIL method, we implement two different SSL approaches, Wang^26^ and Ciga,^27^ as feature extractors. The SSL‐ABMIL method utilizes attention pooling functions to aggregate tile‐level predictions for obtaining WSI‐level predictions. Wang et al.^26^ employed clustering‐guided contrastive learning to train a ResNet‐50 model on 15 million pathological images. Ciga et al.^27^ utilized the SimCLR method to train a ResNet‐18 model on 400,000 pathological images selected from 57 datasets. We extracted 1024 features per image tile using Ciga‐ABMIL and 2048 features using Wang‐ABMIL, based on these pretrained models, forming instance embeddings. A gated attention mechanism automatically calculates attention weights for each instance embedding, and the weighted sum is then combined to form a bag‐level embedding, representing the WSI‐level prediction. The general workflow of this study is illustrated in Figure 1.

### 2.5 Implementation of ImageNet‐MIL and SSL‐ABMIL

Implementation of the ImageNet‐MIL method involved using a ResNet‐50 model pretrained on ImageNet as the feature extractor, with feature aggregation performed using the average pooling function. Hyperparameters were set as follows: batch size of 32, Adam optimizer (β1 = 0.9, β2 = 0.99, ε = 10^−5^), a learning rate of 1 × 10^−3^, and 1% weight decay. The widely used cross‐entropy loss function was employed. The entire training process spanned 50 epochs.

For the implementation of the two SSL‐ABMIL methods, we first embedded the features extracted by ResNet into a 256‐dimensional vector h_k_ using a fully connected layer followed by the ReLU function. As shown in Equation 1 and Figure 1B, each h_k_ was then passed through two parallel fully connected layers (V and U), activated by the tanh and sigmoid functions, respectively. The resulting vectors were dot‐multiplied and mapped to a single value, representing their attention weight (a_k_), using the final fully connected layer (W^T^). The weighted sum of each tile embedding h_k_ and its attention weight a_k_, as depicted in Equation 2, generated a WSI‐level prediction (h_slide_).

(1) $$a_{k}=\frac{\exp\;\left\{W^{T}\left(\tanh\left(Vh_{k}^{T}\right)⊙\text{sigm}\left({\mathrm{Uh}}_{k}^{T}\right)\right)\right\}}{\sum_{j=1}^{K}\exp\;\left\{W^{T}\left(\tanh\left(Vh_{k}^{T}\right)⊙\text{sigm}\left({\mathrm{Uh}}_{k}^{T}\right)\right)\right\}}$$

where $h\in {\mathbb{R}}^{256},V\in {\mathbb{R}}^{128\times 256},W\in {\mathbb{R}}^{256}$ are parameters and K is 512 tiles for each patient.

(2) $$h_{\text{slide}}=\sum_{k=1}^{K}a_{k}h_{k}$$

where *h*_k_ is the k‐th tile's embedding. To obtain the final prediction probability for each patient, *h*_slide_ is passed through a BatchNorm1D layer, followed by a dropout layer with a dropout rate of 30%. Ultimately, *h*_slide_ is fed into a fully connected layer and a softmax layer to obtain the prediction score. The parameters (V, U, W) in ABMIL were learned automatically through training the model, and the hyperparameters were set the same as those in the ImageNet‐MIL method.

### 2.6 Statistical analysis

We trained all deep neural network models (for TMB and VHL mutation) using a five‐fold cross‐validation method in the TCGA cohort, reserving 20% of the dataset as an internal validation set. Subsequently, we applied all models to the external validation CPTAC cohort. The area under the receiver operating characteristic curve (AUROC) was utilized to evaluate the performance of the models. The survival rate difference between high‐ and low‐TMB ccRCC patients was assessed using Kaplan–Meier curve, and a Log‐rank test was conducted. A *p*‐value low than 0.05 was considered statistically significant (two‐tailed).

## 3 RESULTS

### 3.1 Identification of TMB and key mutation

**FIGURE 2:**
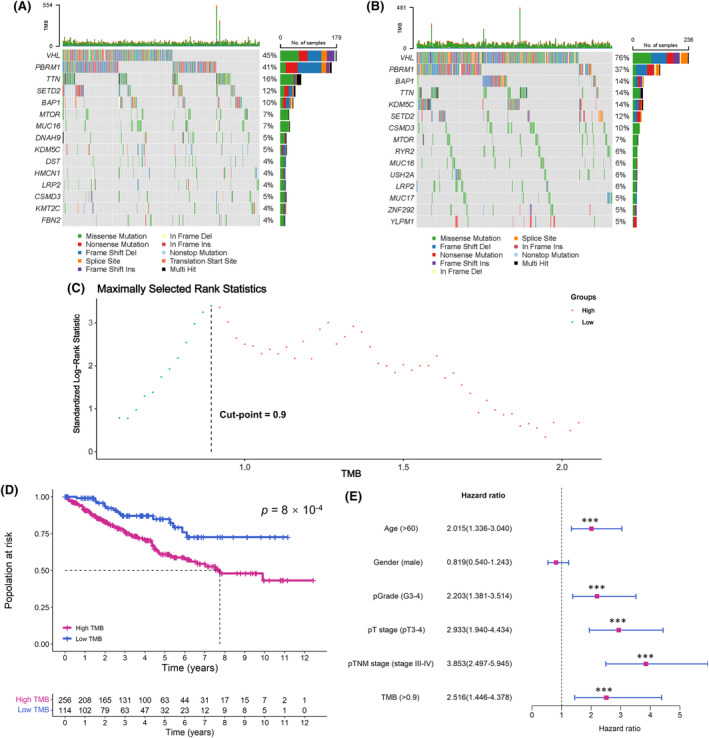
Identification of TMB and VHL mutation. (A, B) represent the gene mutation profiles in the TCGA and CPTAC cohorts, respectively. (C) Calculation of the optimal TMB stratification threshold in the TCGA cohort using the maximally selected rank statistics method. (D, E) Kaplan–Meier curve and Cox regression analysis of the optimal TMB threshold in the TCGA cohort. TMB, tumor mutation burden; TCGA, The Cancer Genome Atlas; CPTAC, Clinical Proteomic Tumor Analysis Consortium. ***p value <0.001.

We calculated the TMB scores based on somatic mutation data from the TCGA and CPTAC databases, as the total number of somatic gene coding errors, base substitutions, gene insertions, or deletions detected per million bases in the entire genome.^29^ Among the two cohorts, VHL was identified as the gene with the highest mutation frequency in ccRCC patients (Figure 2A,B). Subsequently, a threshold of 0.9 was identified as the optimal TMB cut‐point using the maximally selected rank statistics method^30^ in the TCGA cohort, which classified patients into high‐ and low‐TMB groups, and this classification was extended to the CPTAC cohort (Figure 2C). In the TCGA cohort, Kaplan–Meier curve and Cox regression analysis demonstrated that this threshold achieved the best prognostic value (log‐rank *p* = 8 × 10^−4^) (Figure 2D,E).

### 3.2 Performance of ImageNet‐MIL and SSL‐ABMIL in TCGA cohort

**FIGURE 3:**
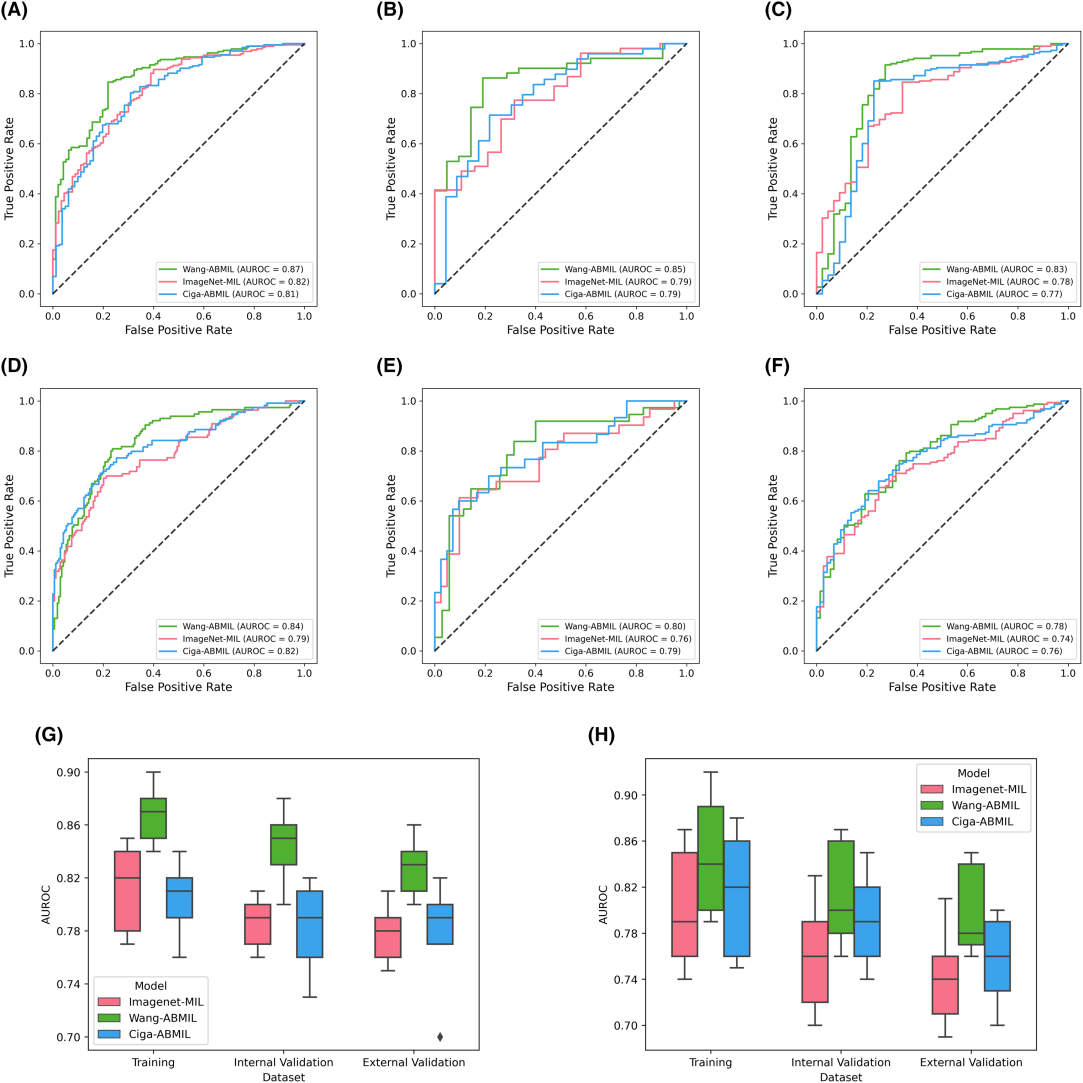
Performance evaluation of deep learning models. (A–C) depict the predictive performance of the three models for TMB on the training set, internal validation set, and external validation set, respectively. (D–F) show the predictive performance of the three models for VHL mutations on the training set, internal validation set, and external validation set, respectively. (G, H) provide a performance comparison of the three models for TMB and VHL mutation prediction tasks. TMB, tumor mutation burden.

First, we trained three models (ImageNet‐MIL, Wang‐ABMIL, and Ciga‐ABMIL) using H&E‐stained histopathological images from the TCGA cohort to assess the predictability of TMB and VHL mutation (Table 2). By comparing the performance of these three models in the prediction task, we found that Wang‐ABMIL achieved the best performance (Figure 3).

**TABLE 2:** Performance results for all models.

Experiment	Target	Cohort	Dataset	Model	Features	AUROC
1	TMB	TCGA	Training	MIL	ImageNet	0.81 ± 0.04
2	TMB	TCGA	Validation	MIL	ImageNet	0.79 ± 0.02
3	TMB	CPATC	Validation	MIL	ImageNet	0.78 ± 0.02
4	VHL	TCGA	Training	MIL	ImageNet	0.80 ± 0.06
5	VHL	TCGA	Validation	MIL	ImageNet	0.76 ± 0.05
6	VHL	CPATC	Validation	MIL	ImageNet	0.74 ± 0.05
7	TMB	TCGA	Training	ABMIL	Wang	0.87 ± 0.02
8	TMB	TCGA	Validation	ABMIL	Wang	0.84 ± 0.03
9	TMB	CPATC	Validation	ABMIL	Wang	0.83 ± 0.02
10	VHL	TCGA	Training	ABMIL	Wang	0.85 ± 0.06
11	VHL	TCGA	Validation	ABMIL	Wang	0.81 ± 0.05
12	VHL	CPATC	Validation	ABMIL	Wang	0.80 ± 0.04
13	TMB	TCGA	Training	ABMIL	Ciga	0.80 ± 0.03
14	TMB	TCGA	Validation	ABMIL	Ciga	0.78 ± 0.04
15	TMB	CPATC	Validation	ABMIL	Ciga	0.76 ± 0.04
16	VHL	TCGA	Training	ABMIL	Ciga	0.81 ± 0.06
17	VHL	TCGA	Validation	ABMIL	Ciga	0.79 ± 0.04
18	VHL	CPATC	Validation	ABMIL	Ciga	0.76 ± 0.04

For the prediction of TMB, Wang‐ABMIL obtained AUROCs of 0.87 ± 0.02 on the training set and 0.84 ± 0.03 on the internal validation set. In comparison, ImageNet‐MIL performed slightly worse with AUROCs of 0.81 ± 0.04 on the training set and 0.79 ± 0.02 on the internal validation set. Ciga‐ABMIL yielded similar results to ImageNet‐MIL, with AUROCs of 0.8 ± 0.03 on the training set and 0.78 ± 0.04 on the internal validation set.

In the prediction task for VHL mutation, both Wang‐ABMIL and Ciga‐ABMIL achieved high performance. Wang‐ABMIL had AUROCs of 0.85 ± 0.06 on the training set and 0.81 ± 0.05 on the internal validation set, while Ciga‐ABMIL had AUROCs of 0.81 ± 0.06 on the training set and 0.79 ± 0.04 on the internal validation set. ImageNet‐MIL performed the worst, with AUROCs of 0.8 ± 0.06 on the training set and 0.76 ± 0.05 on the internal validation set.

### 3.3 Performance of ImageNet‐MIL and SSL‐ABMIL in CPTAC cohort

We further validated the performance of the ImageNet‐MIL and SSL‐ABMIL models in an independent external cohort. For the prediction of TMB and VHL, Wang‐ABMIL achieved AUROCs of 0.83 ± 0.02 and 0.8 ± 0.04, respectively, demonstrating excellent generalizability. In comparison, Ciga‐ABMIL, and ImageNet‐MIL exhibited lower performance in the external cohort. The AUROCs for ImageNet‐MIL were 0.78 ± 0.02 and 0.74 ± 0.05, respectively, while Ciga‐ABMIL achieved both AUROCs of 0.76 ± 0.04. Overall, Wang‐ABMIL demonstrated superior AUROC values and excellent generalization ability in predicting TMB and VHL mutation, displaying high discriminative power and potential clinical relevance.

### 3.4 Attention heatmaps of SSL‐ABMIL with relevant tissue regions

**FIGURE 4:**
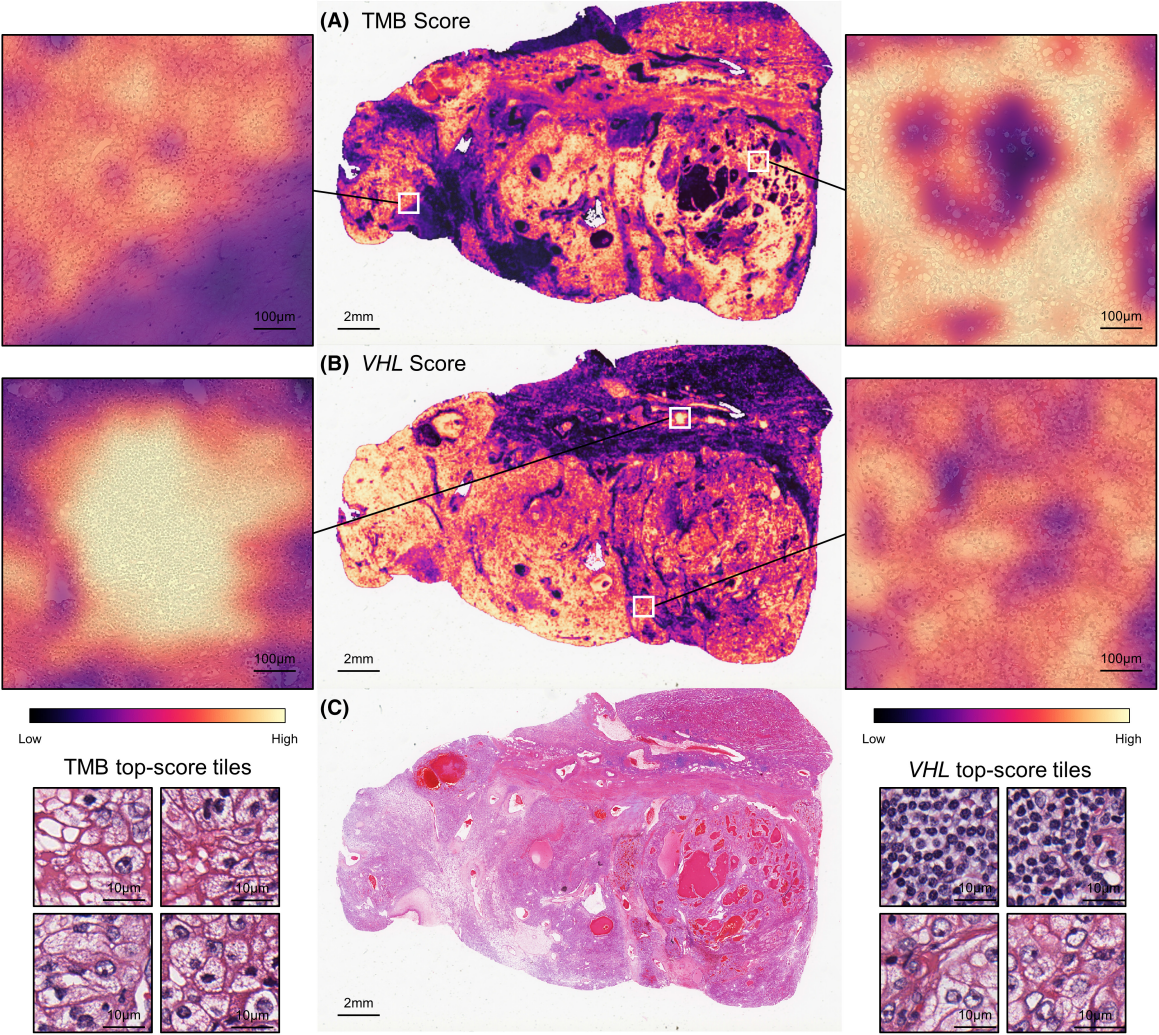
Visualization of attention heatmaps generated by the SSL‐ABMIL model. (A, B) display the heatmap results of the SSL‐ABMIL model for TMB and VHL mutation prediction, respectively. (C) Thumbnail of the original image. TMB, tumor mutation burden; SSL, self‐supervised learning; ABMIL, attention‐based multiple instance learning.

To assist clinicians and pathologists, we visualized the tissue regions of interest identified by SSL‐ABMIL in WSI. With the Wang‐ABMIL model, we can distinguish various regions within the tissue, such as tumor and non‐tumor regions (Figure 4). This was accomplished by leveraging the attention weights and prediction scores generated by the ABMIL model. For TMB prediction, the highly attended areas were mostly limited to the tumor tissue, with less focus on the stromal and necrotic regions (Figure 4A). However, in VHL prediction, the attention of Wang‐ABMIL model became more dispersed. Non‐tumor tissue started to receive attention, but to a lesser degree compared to the tumor tissue (Figure 4B). This suggests that the VHL prediction model may not have effectively learned to fully focus on the tumor tissue or that it discovered pathological features outside the tumor region that are relevant to the prediction. Interestingly, the stromal regions infiltrated by lymphocytes were assigned high attention scores, a phenomenon not observed in TMB prediction. Interfering factors present in WSIs, such as artifacts, may be another influencing factor in VHL mutation status prediction.

## 4 DISCUSSION

In this study, we investigated the ability of DL models to analyze H&E‐stained WSIs for predicting TMB and VHL mutation status in ccRCC, and we validated our results on an independent dataset. We first developed three MIL models based on different feature extractors and successfully explored intratumoral heterogeneity. Wang‐ABMIL model achieved the best predictive performance with AUROCs of 0.87 for TMB and 0.85 for VHL mutation. Furthermore, we examined their performance on an external cohort to demonstrate their decent generalization ability. Effective external validation was lacking for the previous ABMIL method.^25^

DL algorithms can identify and comprehend complex patterns in pathological images, enabling them to discover hidden correlations and patterns within pathology slides. Currently, predicting biomarkers in histopathological slides using DL is a challenging task. Previous studies employed tile‐based deep convolutional neural networks for direct biomarker prediction, but the performance was unsatisfactory. Marostica et al.^18^ constructed a generic DL framework that investigated the mutation status of VHL using only tumor region features and achieved an AUROC of 0.71. Additionally, they directly predicted tumor mutation count, with a Spearman correlation coefficient of 0.419 between the true and predicted values. However, their limitations included tumor region processing, low performance and a lack of external validation, which are fatal for clinical translation of DL models. It should be noted that it is still unknown whether DL models utilize and understand non‐tumor region features, making it inappropriate to rely solely on tumor region features for biomarker prediction. Recently, SSL methods have been applied more extensively in the field of histopathology and have reported higher performance compared to ImageNet‐MIL.^25^ Here, we employed two SSL‐ABMIL methods and demonstrated the superiority of Wang‐ABMIL in predicting biomarkers, consistent with previous studies.^14^, ^25^ In contrast, Ciga‐ABMIL did not achieve satisfactory performance. This may provide evidence that Wang‐encoder trained with clustering‐guided contrastive learning is superior to Ciga‐encoder trained with SimCLR for biomarker prediction. Moreover, our approach only requires WSI‐level labels without the need for region‐level or pixel‐level manual annotations. As histopathological slides obtained in clinical practice do not come with annotated region‐level or pixel‐level labels, the approach is highly applicable.

In recent years, an increasing number of studies have reported that TMB is a promising biomarker, especially in predicting prognosis related to immunotherapy. A minority of somatic mutations in tumor DNA can generate neoantigens, and TMB serves as a useful estimate of tumor neoantigen burden.^34^ TMB can be used to predict the efficacy of immune checkpoint blockade and has become a useful biomarker for identifying patients who will benefit from immunotherapy in many cancer types.^4^, ^5^ Currently, TMB is determined using whole‐exome sequencing, which is not widely employed in routine clinical practice.^35^ Finding more cost‐effective and reliable prediction methods would facilitate the implementation and application of TMB in the clinic. Our SSL‐ABMIL model successfully predicted TMB using histopathological image features, which will facilitate personalized treatment of ccRCC patients in resource‐limited environments, as high‐throughput genomic sequencing is not easily feasible.

Characterizing the intratumoral heterogeneity of ccRCC can aid in determining optimal treatment plans for patients, with the goal of improving their clinical outcomes.^36^ Mutation or methylation of the VHL tumor suppressor gene occur in the majority of ccRCC cases and are often considered as one of the inevitable initial steps in the development of ccRCC.^37^ A recent multinational study involving 943 ccRCC patients demonstrated that tumors with only VHL mutation was associated with significantly improved clinical outcomes.^31^ Patients with VHL mutation accompanied by other driver events should prioritize adjuvant therapy, while those with solely VHL mutation may potentially be spared from further treatment. Alterations in BAP1, PBRM1, and SETD2 are common and important co‐driving factors in tumor development.^38^ Acosta et al.^16^ developed a DL model utilizing H&E‐stained images to predict BAP1, PBRM1, and SETD2 mutations, with only BAP1 achieving higher predictive performance. Here, we successfully predicted VHL alterations, indicating a strong association between morphological features and VHL mutation. Although a decline in model performance was observed in the validation cohort, it was not unexpected. Differences arising from variations in the quality of pathological specimens and staining methods across different cohorts cannot be entirely eliminated.

We further visualized histopathological images with high‐TMB and VHL mutation using an attention mechanism to uncover the relationship between morphological features and genetics. In the high‐TMB group, the Wang‐ABMIL model predominantly focused on the tumor tissue region, while paying less attention to the non‐tumor region. Conversely, in VHL mutation, the model exhibited more dispersed attention, allocating same attention to the non‐tumor region, particularly the stromal area infiltrated by lymphocytes. These morphological features align with previous findings and pathological knowledge.^16^, ^17^, ^39^ These results suggested that H&E‐stained histopathological images of ccRCC contain morphological features that cannot be identified through conventional visual assessment of TMB and VHL. These morphological features are closely associated with patients who may benefit from immune checkpoint inhibitors or other novel therapies.

Despite the advantages of our study mentioned above, limitations exist. One of them is inherent to retrospective studies. The optimal TMB stratification threshold derived from the TCGA cohort may not be applicable to the CPTAC cohort. In the future, it will be necessary to adjust this stratification threshold to a range that can be applied across different cohorts. Another limitation is the imbalance in sample categories used for model training, which might result in unstable model performance. Addressing the impact of class imbalance requires focused attention. It is worth mentioning that our model is trained specifically for VHL somatic mutations, thus its efficacy is limited to predicting somatic mutation types only. VHL loss might also be influenced by epigenetic modifications such as methylation, and their impact on the model should be further considered in the future. Additionally, further efforts are needed to incorporate clinical information and imaging data, among other multi‐omics modalities, to integrate pathological features and collectively enhance the performance.

## 5 CONCLUSIONS

Our study developed a predictive model using H&E‐stained histopathological images to predict TMB and VHL mutation status in ccRCC patients. We demonstrated that the SSL‐based ABMIL model outperformed the traditional ImageNet‐MIL approach, displaying promising results in predicting biological markers. DL played a crucial role in connecting tumor morphology with molecular biology and may offer important diagnostic clues.

## AUTHOR CONTRIBUTIONS

**Qingyuan Zheng:** Investigation (equal); resources (equal); writing – review and editing (equal). **Xinyu Wang:** Investigation (equal); resources (equal); writing – review and editing (equal). **Rui Yang:** Investigation (equal); resources (equal); writing – review and editing (equal). **Junjie Fan:** Investigation (equal); resources (equal); writing – review and editing (equal). **Jingping Yuan:** Investigation (equal); resources (equal); writing – review and editing (equal). **Xiuheng Liu:** Investigation (equal); resources (equal); writing – review and editing (equal). **Lei Wang:** Conceptualization (lead); methodology (equal); visualization (equal); writing – review and editing (equal). **Zhuoni Xiao:** Conceptualization (equal); data curation (lead); formal analysis (lead); methodology (equal); software (lead); visualization (equal); writing – review and editing (equal). **Zhiyuan Chen:** Investigation (equal); resources (equal); supervision (equal); writing – review and editing (equal).

## FUNDING INFORMATION

This work was supported by Hubei Province Key Research and Development Project [grant number 2020BCB051] and Hubei Province Central Guiding Local Science and Technology Development Project [grant number 2022BGE232].

## CONFLICT OF INTEREST STATEMENT

The authors declare that they have no known competing financial interests or personal relationships that could have appeared to influence the work reported in this paper.

## ETHICS APPROVAL AND CONSENT TO PARTICIPATE

The study was conducted in accordance with the Declaration of Helsinki, and approved by the Ethics Committee of Renmin Hospital of Wuhan University. Informed consent was obtained from all subjects involved in the study.

## Supporting information


Data S1.

## Data Availability

The datasets of the TCGA cohort for this study can be found in The Cancer Genome Atlas Program (https://portal.gdc.cancer.gov/, accessed on 15 August 2023) and the CPTAC cohort for this study can be found in the Clinical Proteomic Tumor Analysis Consortium (https://www.cancerimagingarchive.net/histopathology‐imaging‐on‐tcia/, accessed on 15 August 2023).
