# Supplementary material for: Predicting tumor mutation burden and VHL mutation from renal cancer pathology slides with self‐supervised deep learning
Source: Cancer Med. 2024 Aug 21;13(16):e70112. doi: 10.1002/cam4.70112 (PMC11336896; doi:10.1002/cam4.70112)
Supplement: Supplementary file 1 — Data S1. [file CAM4-13-e70112-s001.docx]

**Supplementary Materials**

**Supplementary Table 1a.** **Dataset distribution of patients and corresponding images in the deep learning model.**

|  | Patients | Total images | High-TMB | Low-TMB | VHL Wild | VHL Mutation |
| --- | --- | --- | --- | --- | --- | --- |
| TCGA Cohort | 350 | 356 | 244 | 112 | 213 | 143 |
| CPTAC Cohort | 163 | 232 | 188 | 44 | 73 | 159 |

**Supplementary Table 1b.** **Dataset distribution of images in the training, internal validation, and external validation sets.**

|  | TCGA Cohort | | CPTAC Cohort |
| --- | --- | --- | --- |
|  | Training set | Internal validation set | External validation set |
| ImageNet-MIL | 285 | 71 | 232 |
| Wang-ABMIL | 285 | 71 | 232 |
| Ciga-ABMIL | 285 | 71 | 232 |
